# Assessment of mechanical behavior of sprayed concrete reinforced with waste tire textile fibers

**DOI:** 10.1038/s41598-024-59339-2

**Published:** 2024-04-17

**Authors:** Behzad Khosh, Hadi Atapour

**Affiliations:** Department of Earth Sciences Engineering, Arak University of Technology, Arak, Iran

**Keywords:** Environmental impact, Mechanical properties, Civil engineering

## Abstract

The primary objective of this research is to assess the mechanical properties of shotcrete or sprayed concrete reinforced with waste tire textile fibers (WTTFs). Shotcrete is extensively employed in civil and mining engineering projects for support systems. This study examines the physical and mechanical characteristics of shotcrete samples, both without fibers and reinforced with WTTFs, including density, water absorption, volume of permeable voids, ultrasonic pulse velocity, uniaxial compressive strength, and splitting tensile strength. The reinforced samples consist of WTTF fibers at 0.5%, 1%, and 1.5% concentrations. Test results demonstrate that the inclusion of waste tire textile fibers enhances the mechanical properties of shotcrete, particularly its deformability and energy absorption capacity. Based on the test outcomes, a 1% fiber concentration is recommended as the most suitable ratio for utilizing waste tire textile fibers in shotcrete. Furthermore, these flexible fibers do not impede shotcrete pumping or spraying capabilities.

## Introduction

Sprayed concrete or shotcrete is the general term for a mixture of cement, water, sand, and fine aggregate that is pneumatically projected and dynamically compacted at high velocity onto a surface^1^. Shotcrete applications range from stabilizing rock/soil slopes, supporting tunnels and large underground excavations to repairing existing concrete structures. Today, shotcrete has become a vital material in civil applications due to its several advantages, including variety in shape, good compaction, high strength, durability, good substrate adhesion, and ease of application in difficult access^2,3^.

Since 1900, the year that shotcrete was proposed by Carl Akeley, there have been many developments, both in the techniques and equipment required for the pneumatic spraying of concrete or mortar. In this development path, various additions and admixtures are used to achieve the required properties of the final shotcrete^2–4^. Among the many advances in the shotcrete industry in recent years, one of the most important was the use of different fibers as reinforcements, including steel fiber, polypropylene fiber, polyamide fiber, polyvinyl alcohol fiber, glass fiber, and so on^5,6^. The innovation of using different types of fiber reinforcement in shotcrete has been a significant factor in the increasing trend of shotcrete use.

The main aim of using reinforcing materials such as steel fibers in cementitious materials is to improved their tensile strength, energy absorption, and ductility of cement matrixes^7,8^. Steel fiber, basalt fiber, polypropylene fiber, glass fiber, aluminum fiber, and polyethylene fiber are the common types of fibers used to improve shotcrete and concrete performance^9–11^. Steel fiber-reinforced shotcrete was introduced in the 1970s and has since become an important part of tunneling operations, especially in the United States and Europe^12–15^. Zhang et al.^9^ studied the uniaxial tensile characteristics and stress–strain behaviour of steel fiber-reinforced recycled coarse aggregate shotcrete. The mechanical properties of steel fiber-reinforced concrete in high temperature condition are studied by Li and et al.^16^ and Caballero-Jorna et al.^16^. Liu et al. ^17^ reported that adding polypropylene fibers to shotcrete enhanced tensile strength by improving energy absorption capacity and post-cracking toughness. Several researchers have studied the properties of glass fiber reinforced cementitious mixture. Additionally, natural fibers such as kenaf, flax, and jute were also tested for their influence on concrete and shotcrete performance^18^.

Due to the high growth in the number of cars, the accumulation of large numbers of End-of-Life Tires (ELTs) has become a main waste management problem^19^. Around 1.5 billion tires reach the end of their lives annually worldwide^20^. Therefore, ELTs are a particular type of solid waste generated in large amounts, which their decomposition takes about 600 years^21–24^. Due to the environmental hazards, the Accumulation of ELTs is a growing concern observed in both developing and developed countries^25^. Traditionally, ELTs have been illegally dumped, stockpiled, or landfilled. Nevertheless, none of these ways is a long-term solution. It has been proven that these conventional methods of disposing of ELTs are harmful to the environment and economically burdensome^25^. By 2030, tire use is expected to increase by about 20 percent, generating 1200 million ELTs a year^23,26,27^. On the other hand, to achieve the purposes of the 2030 Agenda for Sustainable Development^28^, while decreasing the consumption of non-renewable resources, increasing investigation has focused on the reuse of wastes in various applications.

In general, ELTs are composed of three main components, including rubber granules, steel wire, and textile fibers^24,29^. The percentage of these components varies depending on the use of tires. Therefore, ELTs may contain approximately 70% rubber granules, 5% to 30% steel wire, and textile fiber up to 15% by weight^30,31^. Rubber granules and steel wires are generally recycled and reused for various applications. Several investigations have been carried out to analyze how varying the rubber content affects the mechanical behavior of sand-rubber mixtures^32–35^. Fu et al. stated that the mechanical properties of sand-rubber mixtures are considerably affected by the proportion of rubber incorporated in the mixtures^32^. Samarakoon et al.^36^ and Liew and Akbar^37^ have investigated the mechanical properties of concrete reinforced by steel fibers recovered from scrap tires, such as compressive strength, toughness, and splitting strength. In the treatment process of ELT, unlike rubber granules and steel wires, textile fibers are separated as a discard. On the other hand, textile fibers are categorized as special waste (under CER-certified emission reduction code 19.12.08) that needs appropriate treatment^30,31^.

Presently, textile fibers obtained from ELTs are mainly landfilled. For example, in Europe, around 320,000 tons of textile fibers are disposed each year^30^. In current years, researchers in different scientific fields are trying to put an end to the recent operation of landfilling and therefore to reuse these recycled fibers^31,38–43^. Previous investigations have focused on alternative applications of waste tire textile fibers, for example, additives for sound-absorbing materials, cementitious composites, plastic materials, reinforcement of soil, and so on^22,23,30,44–47^. Based on the literature review, there is very limited research on the usage of WTTF fibers in shotcrete. The authors' initial laboratory research has revealed the potential utility of these fibers in enhancing cementitious material properties^48^. In the present study, in order to evaluate the impact of waste tire textile fibers on dry-sprayed shotcrete, a comprehensive investigation has been conducted on physical and mechanical properties of WTTF fibers reinforced under real field conditions. The research aims to assess the performance of WTTF fibers reinforced shotcrete by examining its density, water absorption, volume of permeable voids, Uniaxial Compressive Strength (UCS), Splitting Tensile Strength (STS), and Ultrasonic Pulse Velocity (UPV). In addition, one of the main challenges in fiber-reinforced shotcrete technology is determining the optimal percentage of fibers. In the present study, in order to find the optimum composition of WTTF fiber in shotcrete, the performance of fiber reinforced shotcrete with different dosages of WTTF has been investigated. The obtained results represent an important step forward in understanding the practical application of WTTF fibers in shotcrete technology.

## Materials and methods

This research study aims to investigate the impact of WTTF fiber on both the physical and mechanical properties of shotcrete. To achieve this objective, a series of experiments were conducted on sprayed specimens. These experiments included tests to determine density, water absorption, porosity, ultrasonic pulse velocity, uniaxial compressive strength, and splitting tensile strength. Additionally, the study aimed to identify an optimal combination of mixtures that would enhance the overall performance of shotcrete for practical applications. This involved analyzing various mixtures in order to determine the most effective combination that would yield better results. In the following, the materials used and the study method are explained.

### Materials

The shotcrete specimens were created using Portland cement, natural aggregate, and WTTF fibers. The specific composition of the mixtures, including the precise amount of fibers required to produce one cubic meter of shotcrete, is reported in Table 1. Different quantities of WTTF fiber were used. A water to cement ratio of 0.45 was maintained throughout the experimentation process.

**Table 1:** The composition of the mixtures.

Mix no.	Fiber content (%)	Aggregate (kg/m^3^)	Cement (kg/m^3^)	Fiber (kg/m^3^)	Water (kg/m^3^)	W/C
1	0	1750	400	0	180	0.45
2	0.5	1750	400	11.65	180	0.45
3	1	1750	400	23.30	180	0.45
4	1.5	1750	400	34.95	180	0.45

#### Cement

Portland cement (type II) according to ASTM C150^49^ produced by Saman-Gharb Cement Group was used as the binder in the present study. Table 2 presents the chemical composition, mineralogical, mechanical, and physical properties of the cement, as provided by the manufacturer.

**Table 2:** Chemical composition, mineralogical and physical properties of cement used in this research.

Chemical composition (%)		Mineralogical composition (%)		Physical and mechanical properties	
SiO_2_	20.7 ± 0.3	C_3_S	59.47	Blaine (cm^2^/g)	3200 ± 100
Al_2_O_3_	5.2 ± 0.2	C_2_S	14.48	Auto clave method (%)	0.08 ± 0.02
Fe_2_O_3_	4.6 ± 0.2	C_3_A	6	Initial setting time (min)	140 ± 20
CaO	65 ± 0.5	C_4_AF	14	Final setting time (min)	240 ± 20
MgO	1.8 ± 0.2			3-day compressive strength (kg/cm^2^)	270 ± 20
SO_3_	2.2 ± 0.4			7-day compressive strength (kg/cm^2^)	440 ± 20
K_2_O	0.5 ± 0.05			28-day compressive strength (kg/cm^2^)	530 ± 20
Na_2_O	0.15 ± 0.05			Sieve 0.09 mm	1.1 ± 0.1
L.O.I	1 ± 0.5			Density (gr/cm^3^)	3.12 ± 0.01
I.R	0.4 ± 0.1				
Free CaO	1.3 ± 0.2				

#### Water

The tap water was used as the mixing water to produce shotcrete for specimen preparation, which meets the ASTM C1602 standard specification for concrete mixing water^50^.

#### Aggregate

The grain size and distribution, grain type (natural river or crushed sand), and grain properties directly affect the quality of shotcrete. The European standard, EN 12620^51^, and the American Society for Testing and Materials, ASTM C1436^52^, for aggregate, were followed in this research. In this study, natural carbonate-based aggregate was used.

**Figure 1:**
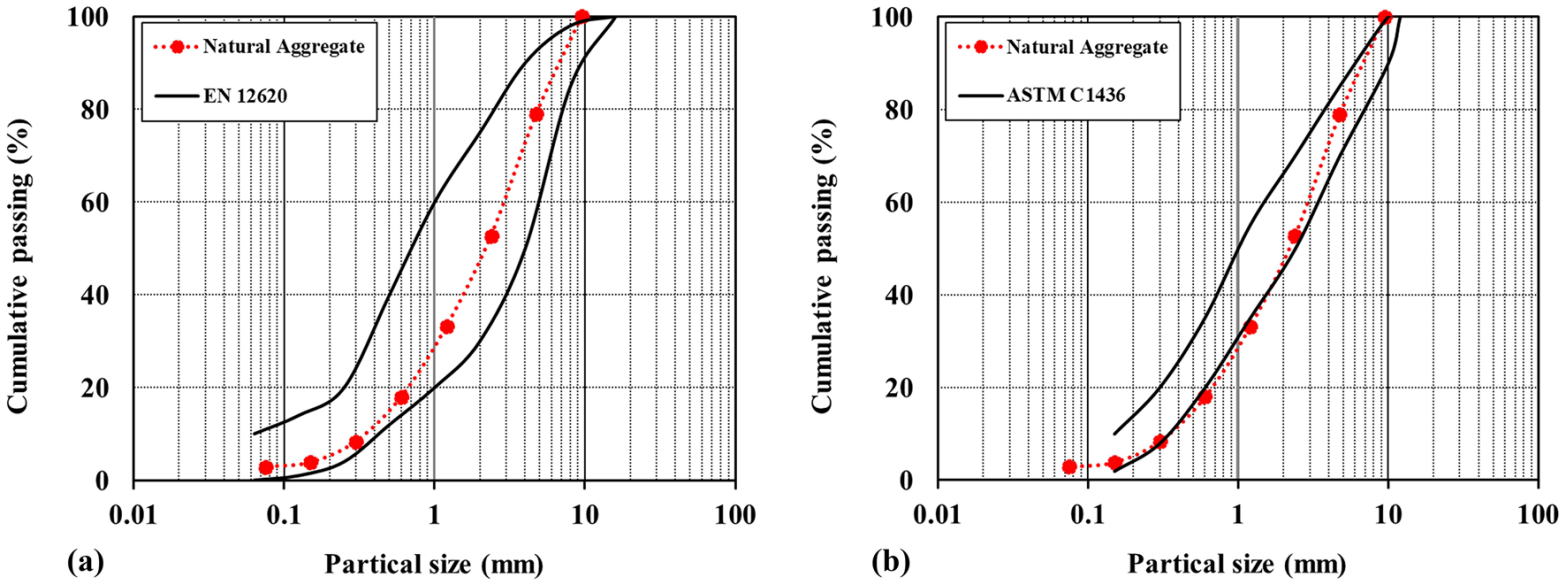
Sieve analysis curves of natural river aggregate along with the (**a**) EN 12620 standard^51^ (**b**) ASTM C1436 standard (ASTM C1436, 2013).

Figure 1a illustrates the natural aggregates sieve analysis curves along with the European standard curves. As shown in this figure, both aggregates are within the EN 12620 standard range^53^ and are approved in terms of grain size and grain distribution. In addition, Fig. 1b shows aggregate grading curves along with the standard curves provided by ASTM. According to ASTM C1436^52^, the aggregate curve is within the allowable range. Considering the natural aggregate grading curves in both standards, finally, this aggregate was used to produce fiber-reinforced shotcrete in the present study. The size of used aggregates is in the range of 0 to 9.5 mm.

The physical properties of natural aggregates, including density, moisture content, sand equivalent value, materials finer than 75-μm (No.200) sieve, and water absorption, were determined using standard test methods. The physical properties of the used aggregate are summarized in Table 3.

**Table 3:** Physical properties of the used aggregate.

Property	Unit	Values	Testing method
Density (SSD)	kg/m^3^	2569	^54^
Moisture content	%	4.38	^55^
Sand equivalent value	%	81	^56^
Fineness modulus (fm)	–	3.7	^57^
Water absorption	%	3.95	^54^
Passing No.200 (75µm) sieve	%	2.29	^58^
Coefficient of uniformity (C_u_)	–	6	^59^
Coefficient of curvature (C_c_)	–	1	^59^

#### Waste tire textile fiber

**Figure 2:**
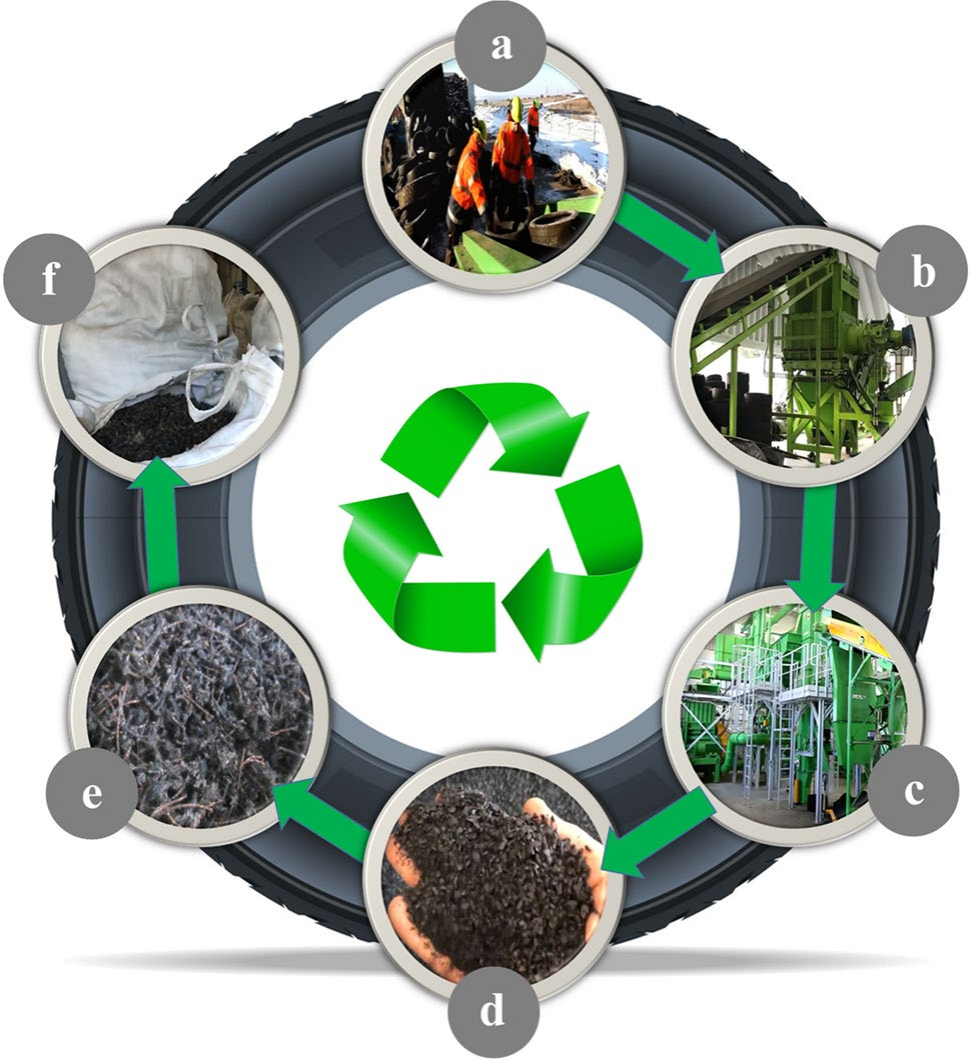
Treatment process of end-of-life-tires, (**a**) gathering the ELTs, (**b**) shredding operation, (**c**) separating the generated components by applying suction, (**d**) rubber powder, (**e**) waste tire textile fiber, (**f**) compacting and packing the WTTFs.

In this research, waste tire textile fibers (WTTF) were sourced from the recycling of End-of-Life Tires at the Yazd Tire factory. This factory produces a daily waste of approximately 5 to 8 tons of WTTF fibers, which are traditionally disposed of in landfills. The process of ELTs treatment and WTTF acquisition is illustrated in Fig. 2. It involves collecting the ELTs, shredding them into smaller particles using a mechanical regular temperature shredding technique, separating the resulting components via suction to obtain rubber powder and WTTFs, and finally compacting and packaging the WTTFs.

**Figure 3:**
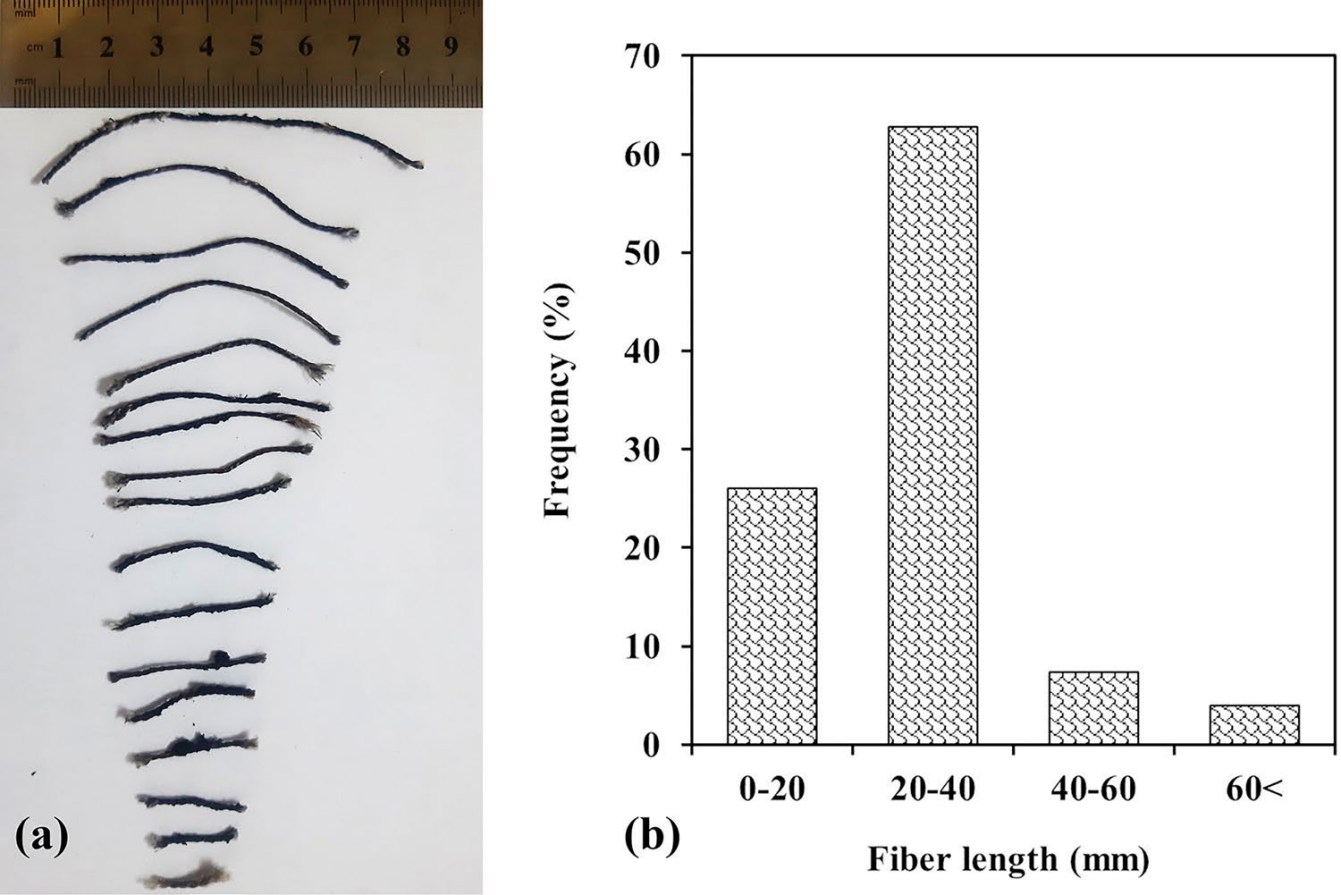
(**a**) Longitudinal shape of the used WTTFs; (**b**) length distribution analysis of used WTTFs.

Since the factory entrance contains different types of vehicle tires, the resulting textile fibers have variable dimensions (diameters and lengths) and mechanical properties. Figure 3a shows how the longitudinal shape of the WTTF differs from that of ordinary industrial fibers. The results of the length distribution analysis of the textile fibers used in the present study are shown in Fig. 3b As illustrated in this figure, WTTF fibers range in length from 5 to more than 80 mm, with about 63% of the fibers being between 20 and 40 mm long and less than 5% being longer than 60 mm. These lengths are typical of the fibers used in the shotcrete industry. For example, the lengths of the polyamide and steel fibers used by Guler et al.^60^ were 54 and 60 mm, respectively. Among the textile fibers, some rubber particles can be seen that are attached to the fibers. The received textile fibers were used without supplementary sorting or cleaning.

The average physical and mechanical properties of WTTF fiber are presented in Table 4. These results were obtained from tests carried out on WTTF fiber in the technical department of Yazd Tire Factory. The main constituent material of textile fiber is nylon, particularly nylon 6/6. In the present study, several dosages of WTTF fiber, including 11.65 kg/m^3^, 23.3 kg/m^3^, and 34.95 kg/m^3^, were used in shotcrete, which were equivalent to mass ratios of 0.5, 1, and 1.5%.

**Table 4:** Physical and mechanical properties of WTTFs used in this research.

Property	Unit	Values		Testing method
		Range	Most frequency	
Tensile strength	MPa	300–2000	600	^61^
Modulus of elasticity	GPa	2–7.5	2.7	^61^
Length	mm	0–80	20–40	^61^
Diameter	mm	0.035–1.5	0.8	^61^
Elongation	%	18–25	22	^61^
Linear density	Denier*	840–1890	1260	^61^
Melt point	°C	250–260	256	^61^
Hot air shrinkage (at 177 °C × 2 min × 143 g)	%	3–5	4.5	^62^
Water absorption	%	5–13	9.5	^61^
Acid and alkaline resistance	–	Excellent		^61^

*9 km yarn mass in grams.

### Method

#### Mix design and specimen preparation

In this study, the guidelines provided by the ACI 506R^63^ and ACI 506.1R^64^ were used to prepare the mix design for plain and fiber-reinforced shotcretes, respectively. Four different sets of specimens were prepared for testing purposes (Table 1). A water to cement ratio of 0.45 was adopted. It is important to note that the cement and aggregate proportions remained consistent across all four sets of specimens. One set of samples was prepared without the inclusion of any fibers, representing a plain mixture. The other three sets were prepared with varying percentages by weight of WTTF fibers: 0.5%, 1%, and 1.5%. The production of fiber-reinforced shotcrete samples requires a significant amount of mixed materials and specialized industrial spraying facilities. In this study, the dry-mix shotcrete method was employed, utilizing industrial equipment located at the Bagan–Mariwan Tunnel site.

**Figure 4:**
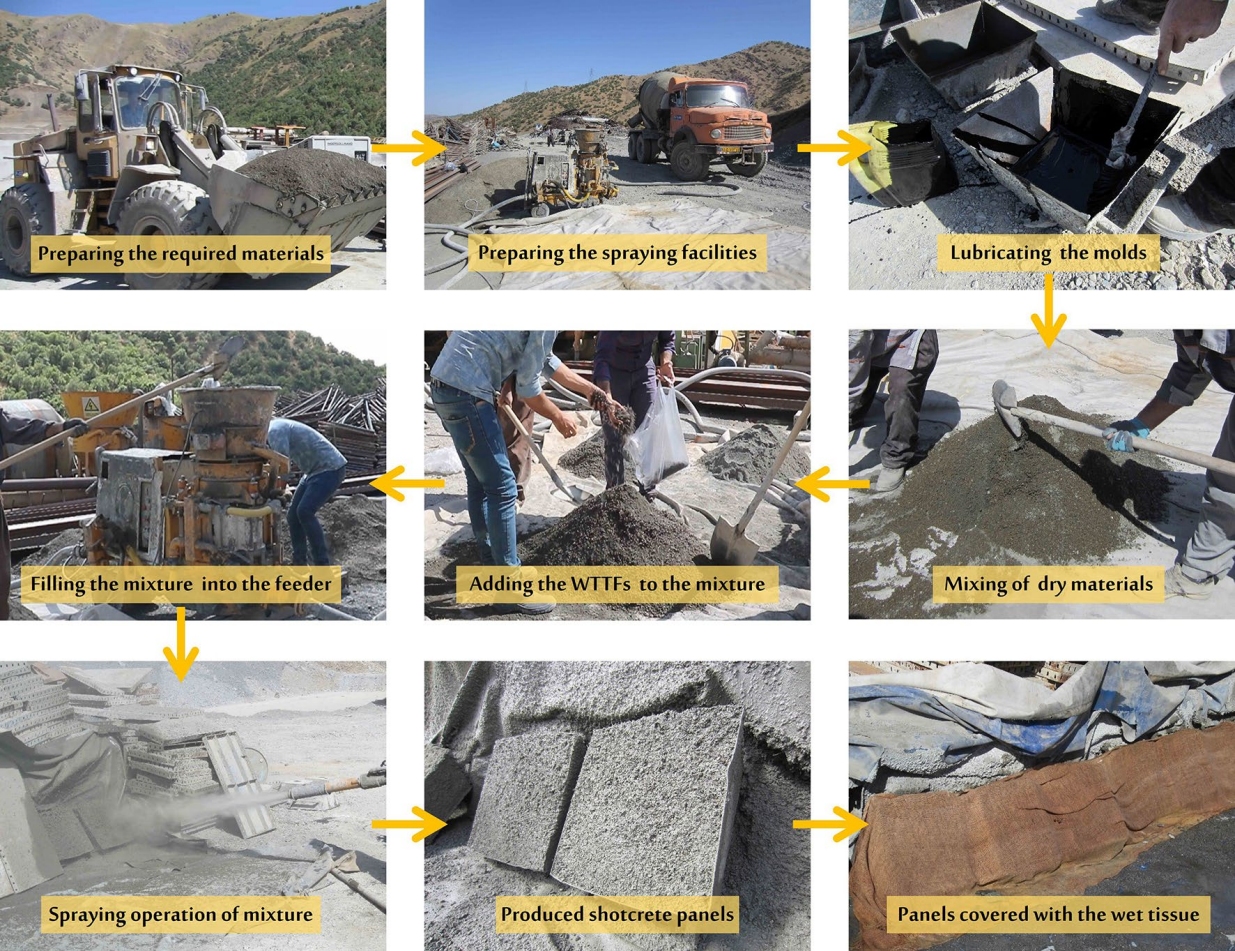
Shotcrete specimen production operations at the site of the Bagan–Mariwan Tunnel project.

Figure 4 provides a visual representation of the various stages involved in the production of shotcrete samples, including:

1. Preparation of materials and equipment: All the necessary materials and equipment, including spraying facilities and molds, were transported to the production site. 2. Dry Mixing: All dry components were carefully mixed. During this mixing process, the WTTF fibers were evenly distributed throughout the materials to ensure homogeneity. 3. Feeding the Mixture: The prepared dry mixture was then fed into the feeder. This feeder conveyed the constituent materials through a delivery hose towards the nozzle body. 4. Mixing with Water: In the nozzle body, water was introduced under pressure using a water ring. It was thoroughly mixed with the other components of the mixture to achieve a consistent composition. 5. Spraying onto Molds: The mixed shotcrete was sprayed through the nozzle onto molds. The dimensions of these molds were approximately 420 mm (length), 420 mm (width), and 200 mm (depth).

The shotcreting operation was controlled by a skilled nozzle man. The spraying direction was perpendicular to the steel mold surface and the nozzle tip was maintained at a distance of about 1.5 m from the mold. These stages collectively allowed for the production of shotcrete samples in a controlled and efficient manner.

**Figure 5:**
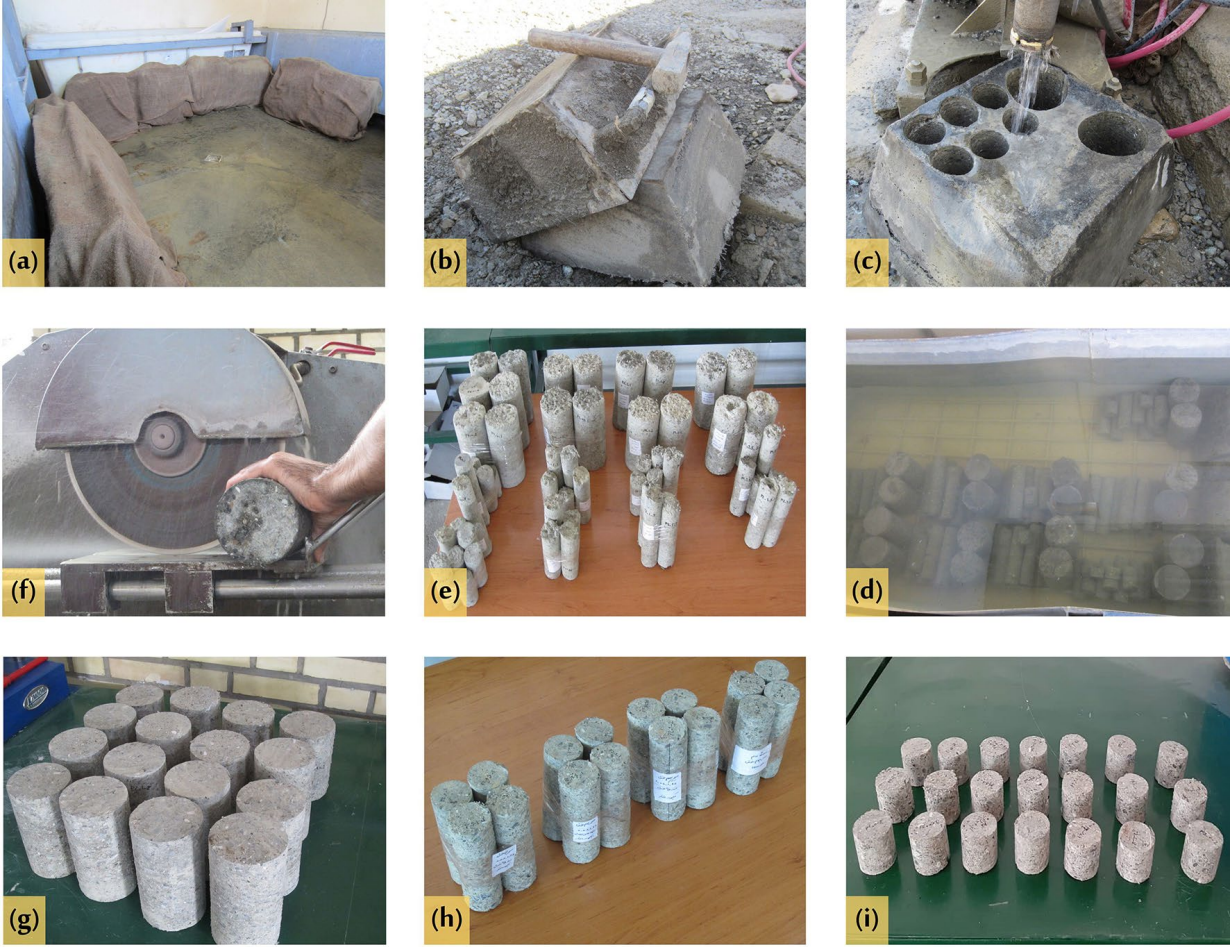
Sample preparation operations, (**a**) tissue-covered panels in curing room, (**b**) removing the panels from molds, (**c**) samples coring operation, (**d**) immersing the samples in water, (**e**) transferring the samples to the laboratory, (**f**) cutting the samples end, (**g**) prepared samples for uniaxial compressive strength and ultrasonic pulse velocity tests, (**h**) prepared samples for physical properties tests, (**i**) prepared samples for splitting tensile strength tests.

On the same day, all four designed mixes were shot using the specified procedure. Three panels were prepared using the plain mix without any fibers, while the other mixtures were used to prepare two panels each. Immediately after the shotcreting operation, all filled molds were covered with completely wet tissue. This step was taken to minimize evaporation and ensure proper hydration of the shotcrete. After a 24-h period, the molds were transferred to a curing room (Fig. 5). In the curing room, the panels were again covered with wet tissue and periodically moistened for a duration of one week. The temperature in the curing room was set to 25 ± 2 °C to maintain optimal curing conditions. At the end of the one-week curing period, the panels were removed from the molds. From these hardened panels, designed samples were obtained by coring cylinders. Each panel yielded cylindrical samples with diameters of 100 mm and 54 mm (NX core). Subsequently, all the obtained samples were immersed in water for a period of three weeks.

#### Test procedures

Experimental investigations were conducted to assess the quality of WTTF fiber reinforced shotcrete through physical and mechanical property tests. The physical tests focused on determining the density, water absorption, and volume of permeable voids. Meanwhile, the mechanical tests included measurements of uniaxial compressive strength, splitting tensile strength, and ultrasonic pulse velocity. These tests were performed on various sizes of cylindrical samples. The detailed test methods are describded below.

##### Density, water absorption, and volume of permeable voids

**Figure 6:**
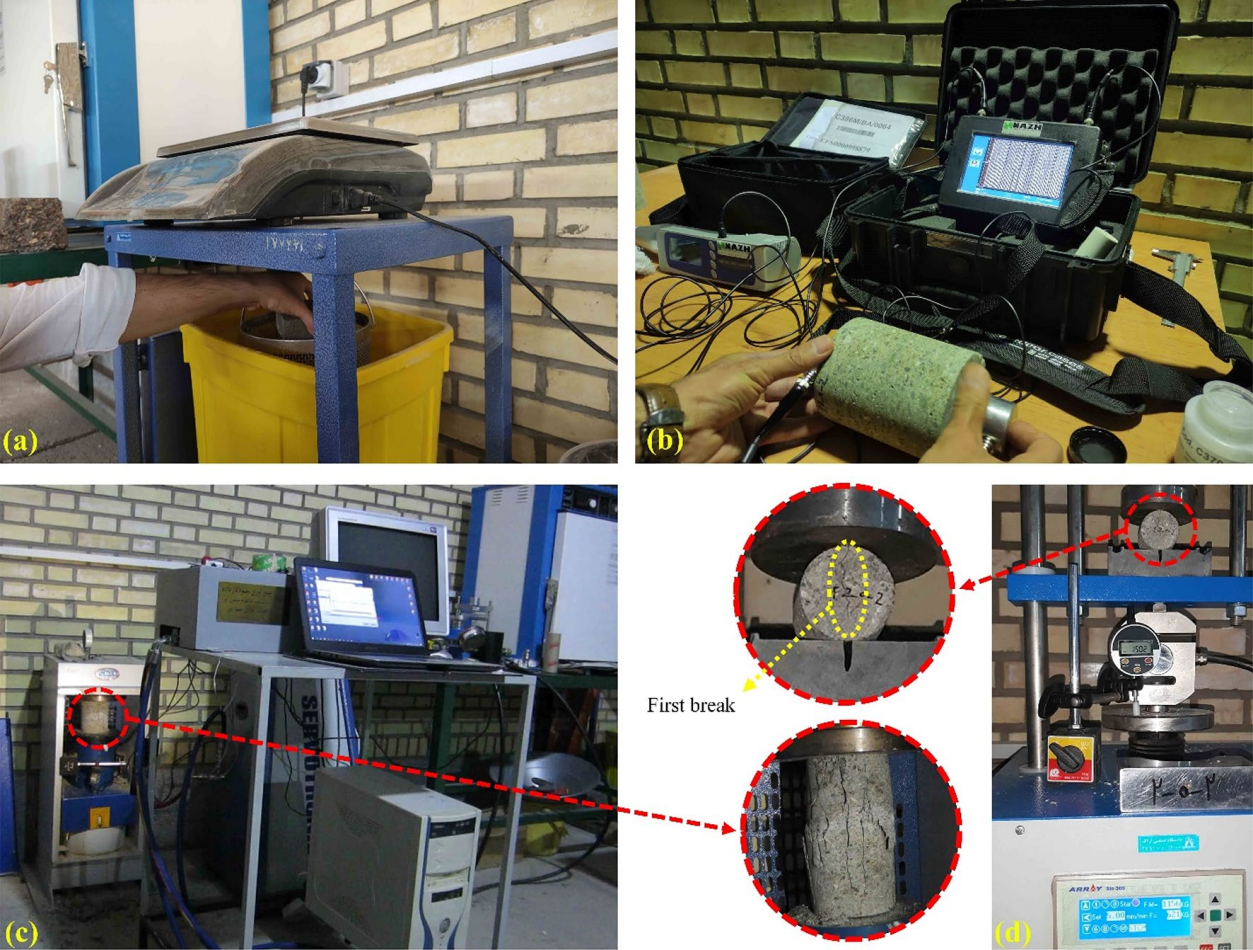
A view of the setup and testing methods, (**a**) physical propertie tests, (**b**) ultrasonic pulse velocity tests, (**c**) uniaxial compressive strength tests, (**d**) splitting tensile strength tests.

In the shotcrete industry, density, void content, and water absorption are used as quality control indicators. Density, also recognized as unit weight, is very important in the shotcrete design process. The test procedures described in ASTM C642^65^ were used to determine the density, void content, and water absorption of plain and WTTF fiber-reinforced shotcrete. In the first stage, the specimens were oven-dried at 105 °C. The weight of the samples was determined at 24-h intervals, to the extent that the difference in mass of two sequential measurements was less than 0.5%. Subsequently, the specimens were immersed in water at a temperature of about 25 °C for 48 h so that the mass equilibrium between the two measurements reached 0.5% at 24-h intervals, and then their saturation mass was measured (Fig. 6a).

##### Ultrasonic pulse velocity

Ultrasonic pulse velocity is a common, simple, and economical non-destructive technique to determine material properties. This test method can be used to evaluate the uniformity and relative quality of rock or cement base materials in both laboratory and field conditions^66,67^. As illustrated in Fig. 6b, the UPV tests were carried out on cylindrical specimens per ASTM C597^68^ and using the ultrasonic pulse velocity apparatus (MATEST Company). The frequency of the used transducers was 55 kHz. Since the values of ultrasonic pulse velocity are highly affected by the size and shape of the specimen, all the cylindrical specimens were prepared at the same size. The diameter of the core specimens for the UPV test was 100 mm, and the length of the specimens was 105 mm. The quality of the transducer-specimen coupling is very important for the accuracy of the technique. Grease, as an appropriate coupling paste, was applied to reduce the effect of the void between the transducer and the specimen.

Pulses of compressional waves were generated by an electro-acoustic transducer that was attached to the one flat end of the specimen under test. After passing through the specimen, the pulses were received by the second transducer, which was located at the other flat end of the specimen, and were converted into electrical energy (Fig. 6b). Thus, the ultrasonic pulse transit time pulse to travel along the specimen was measured. The values of UPV were then calculated by dividing the specimen length (m) per pulse transit time (s).

##### Uniaxial compressive strength

In most construction projects, uniaxial compressive strength is the most common test for mechanical property investigation of shotcrete. The uniaxial compressive strength of plain and WTTF reinforced shotcrete specimens was determined using ASTM C39^69^. To perform UCS tests, cylinder samples with a diameter of 100 mm and a length of 200 mm were used. For each mixture, prepared specimens were tested after 28 days of curing to determine the average UCS. All tests were performed using a 2000 kN hydraulic compression testing machine with a constant rate of loading equal to 1 mm/min (Fig. 6c). Given this loading rate, each experiment lasted about 10 min.

##### Splitting tensile strength

The splitting tensile strength of specimens was obtained according to the ASTM C496^70^ on the 28th day. To determine the average value of STS, the cylindrical specimens were tested per mixture, as shown in Fig. 6d. The tests were performed at a constant rate of 5 mm/min. Loads and deformations were recorded at resolutions of 0.01 kN and 0.001 mm, respectively.

## Results and discussion

### Density, water absorption, and volume of permeable voids

**Figure 7:**
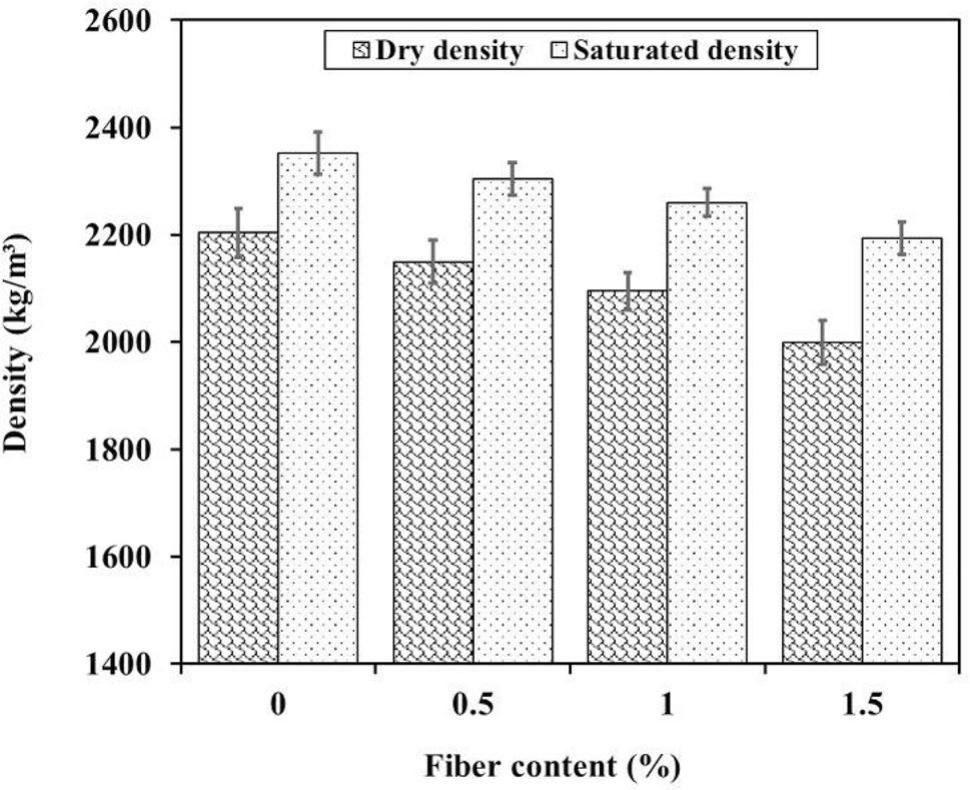
Oven-dry and saturated density of samples vs. WTTFs content.

The density of shotcrete is influenced by a variety of factors including aggregate density, void content, cement content, and water content in the mixture design. Figure 7 displays the average values of oven-dry and saturated densities of plain and fiber reinforced shotcrete, which were determined at 28 days. As shown in the figure, the values vary depending on the fiber proportion in each mixture. The dry and saturated densities of the samples have decreased as the WTTF fiber proportion in the shotcrete mixture has increased, due to the lower unit weight of WTTF when compared to other shotcrete components. As WTTF fibers are relatively lightweight, their incorporation into the shotcrete mixture reduces the proportion of heavier components, leading to a decrease in the density of the overall composite material. In addition, the rough surfaces of WTTF fibers have a tendency to trap air, which also contributes to the reduction of mixture density. Therefore, increasing the proportion of WTTF fibers in the mixture decreases the density of shotcrete by increasing the void content. The range of dry density for the mixtures was between 1999 to 2203 kg/m^3^, with variations of approximately 10%. This indicates that WTTF has a significant influence on shotcrete density. Decreasing the density can simplify the shotcrete operation; additionally, thicker layers of shotcrete can be applied during each spraying step.

**Figure 8:**
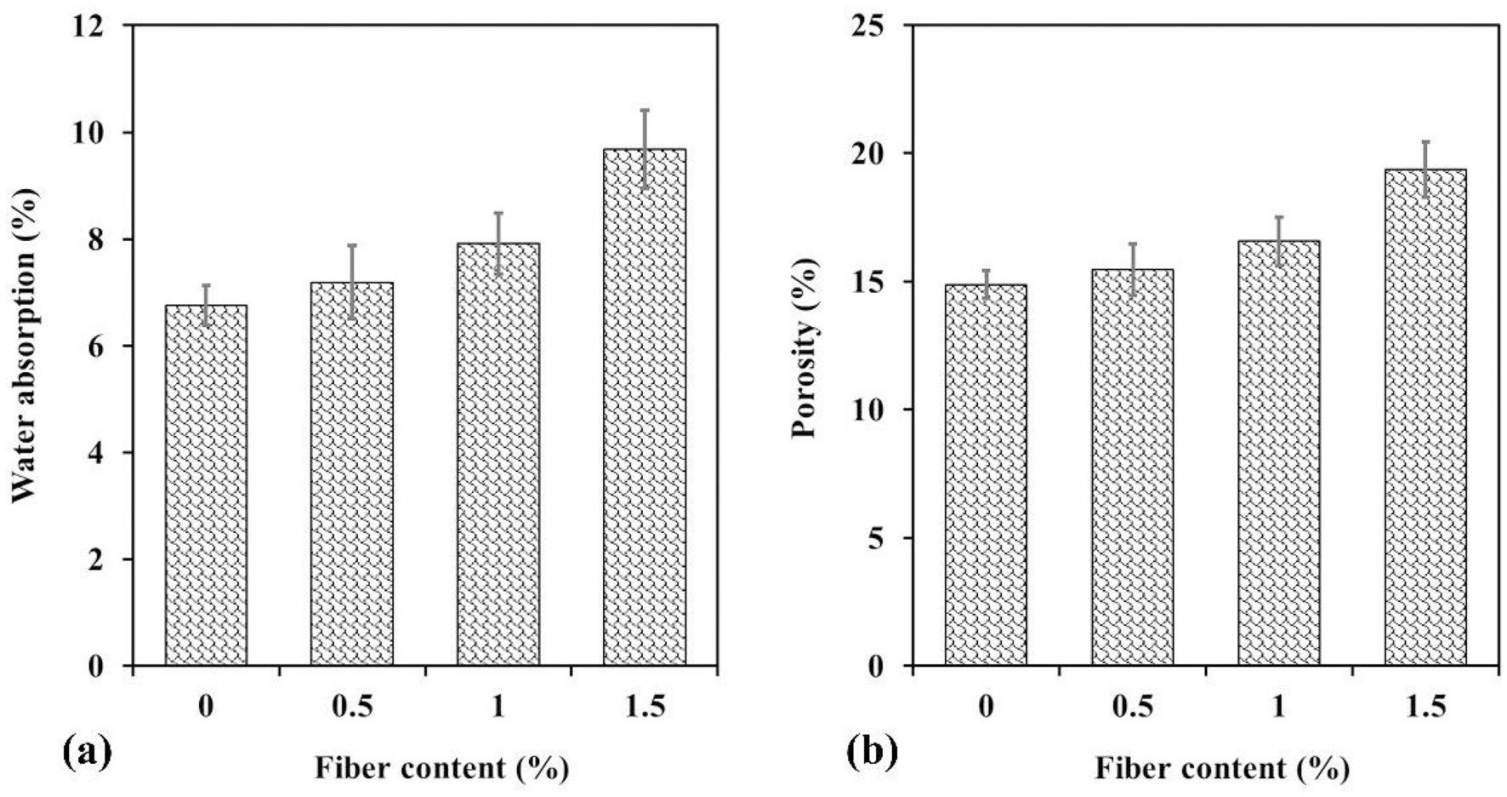
(**a**) Water absorption values of samples vs. WTTFs content, (**b**) porosity values of samples vs. WTTFs content.

In Fig. 8a, water absorption values of all samples in 28 days are shown. The results show that the increase in WTTF fiber content provided a considerable increase in the water absorption values. The lowest value was obtained in plain shotcrete (equal to 6.75%) and the highest value was obtained in 1.5% WTTF fiber-reinforced shotcrete (equal to 9.68%). There is an increase of about 43% in water absorption with the addition of 1.5% WTTF fibers compared to the fiber-free sample. The increase in water absorption may be attributed to the formation of more void volume between WTTF fibers and the cement paste in the shotcrete mixture. This was verified by the porosity values in Fig. 8b. As shown in this figure, the highest porosity, equal to 19.35%, was obtained in the 1.5% WTTF fiber reinforced shotcrete, which also yielded the highest value of water absorption. This is while the porosity of fiber-free shotcrete was equal to 14.87%. The differences are about 30%, which means that the increase in WTTF fiber ratio considerably affects the porosity values. The increasing void content of shotcrete samples with WTTF fiber additive is due to the higher porosity of the tire textile fibers than the sand aggregates and the very porous surface of these fibers. In other studies, similar results have been reported. Al-Owaisy^71^ showed that the amount of voids increases with the percentage of steel fibers. Moreover, Gebretsadik et al.^72^ presented that fibers considerably contribute to the formation of voids. Because the presence of fibers prevents the mix from moving freely, additional void spaces are generated. As shown in Fig. 8, the water absorption and porosity variations with fiber content are fairly comparable. Because there is a direct relationship between porosity and water absorption, more water is absorbed by voids as porosity increases.

### Ultrasonic pulse velocity

**Figure 9:**
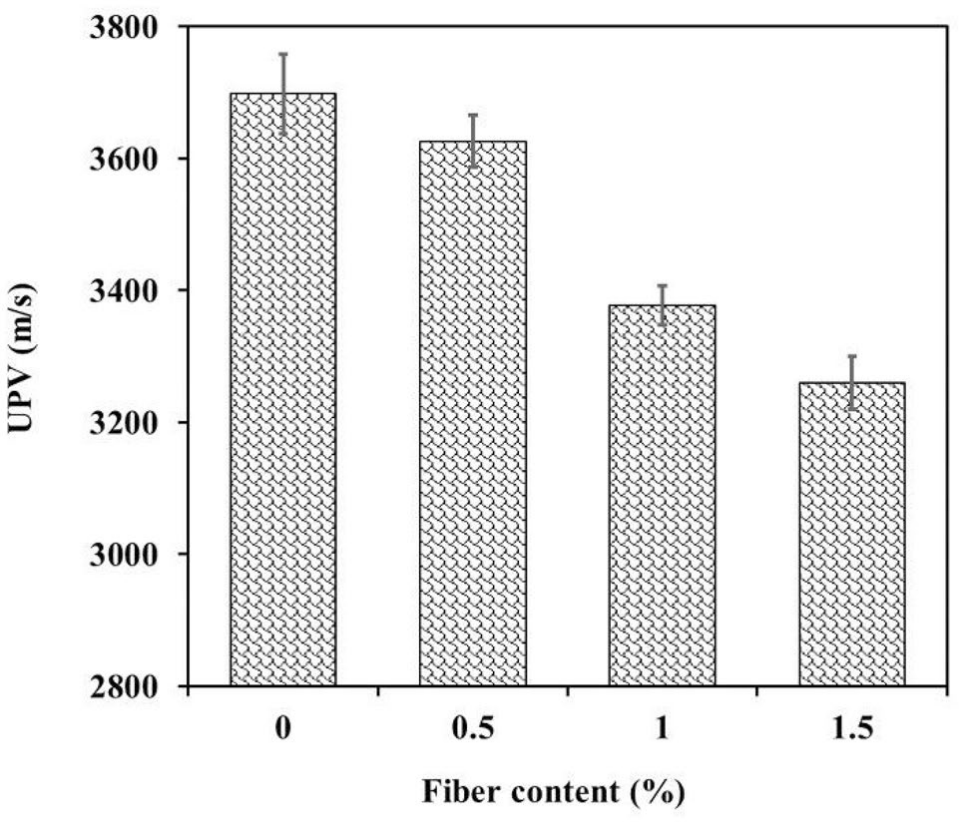
Effect of fiber ratio on ultrasonic pulse velocity values.

The relationship between the WTTF fiber incorporation ratio and ultrasonic pulse velocity is presented in Fig. 9. As seen in this figure, there is a strong relationship between these parameters. The ultrasonic pulse velocity was lower in the mixtures that contained more WTTF fibers. As the fiber content increased by 0.5%, 1%, and 1.5%, the ultrasonic pulse velocity decreased by 1.9%, 9.5%, and 13.4%, respectively. This behavior is not unexpected and can be related to the following causes: The values of the ultrasonic pulse velocity are controlled by the material's density and porosity. Since the ultrasonic pulse velocity in low density WTTF fibers is lower than other shotcrete components, i.e. aggregate and cement, the ultrasonic pulse velocity is expected to decrease as the proportion of fibers increases. Furthermore, the porosity of the mixtures has increased with increasing fiber content, so increasing the porosity is another reason for the decrease in ultrasonic pulse velocity in the WTTF fiber reinforced shotcrete samples. Similar findings on the relationship between ultrasonic pulse velocity and fiber content have been presented by other researchers^72^.

**Figure 10:**
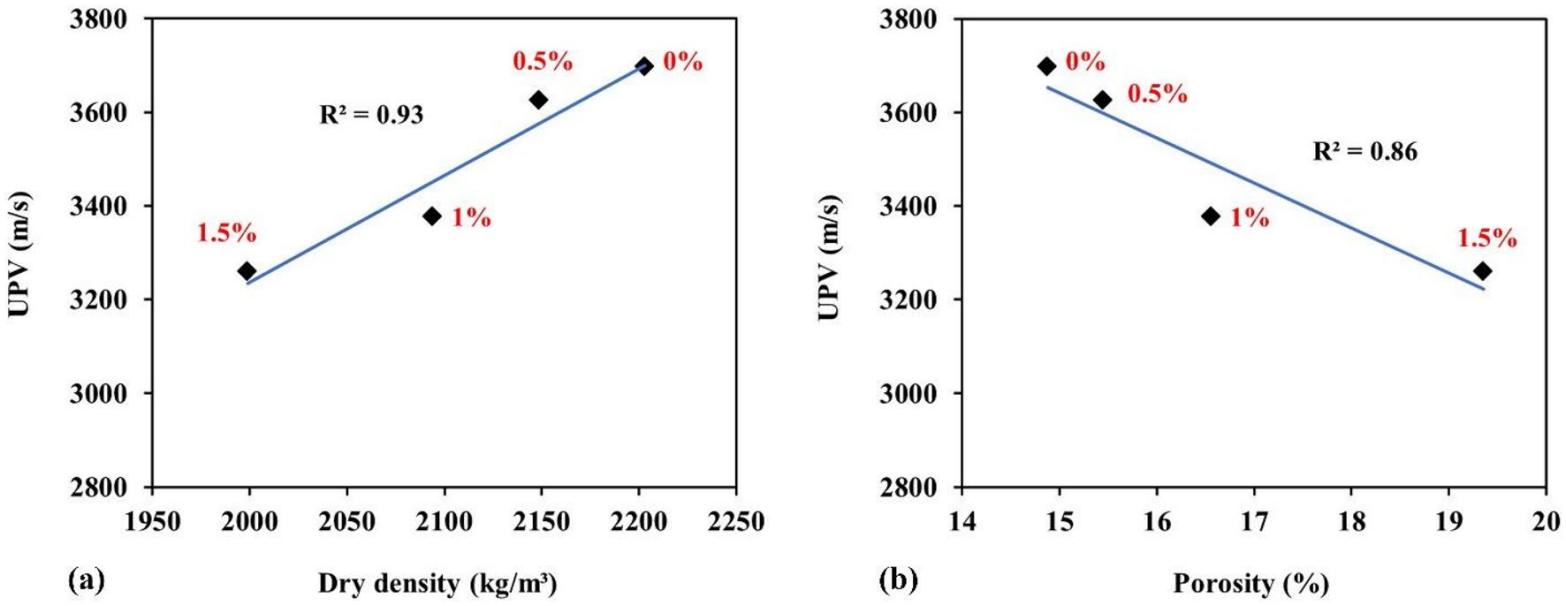
The relations between (**a**) ultrasonic pulse velocity and density and (**b**) ultrasonic pulse velocity and porosity.

The relationships of ultrasonic pulse velocity with density and porosity were analyzed, as illustrated in Fig. 10a and b, respectively. These parameters are intrinsically related. As seen in Fig. 10, there are obvious relations between UPV and density and porosity, with R^2^ values above 0.93 and 0.86, respectively, which is consistent with the fact that decreasing density and increasing porosity contribute to the reduction of ultrasonic pulse velocity.

### Uniaxial compressive strength

**Figure 11:**
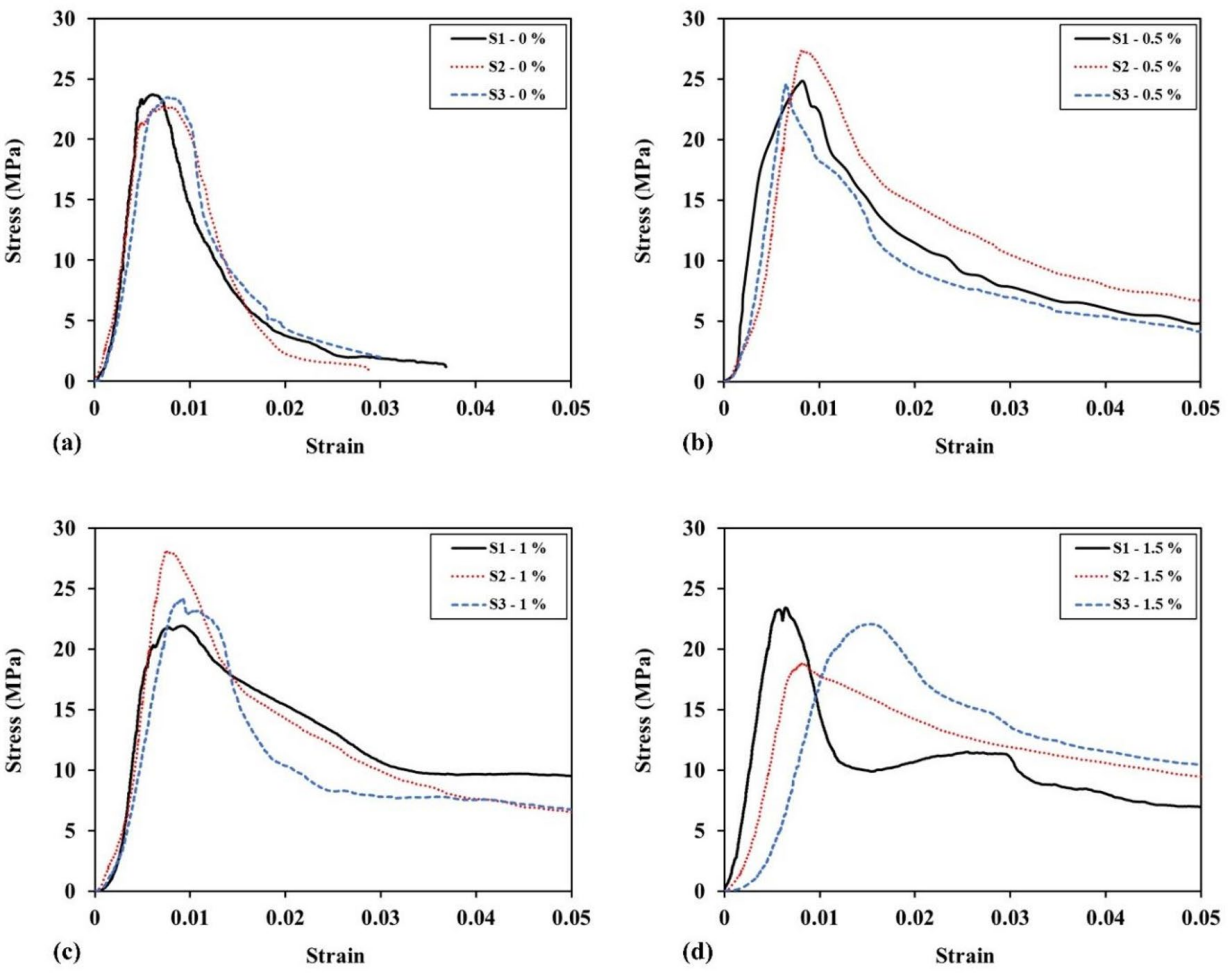
Stress–strain curves of uniaxial compressive strength tests with different WTTFs ratio.

The stress–strain curves for plain shotcrete specimens (Fig. 11a) showed an average uniaxial compressive strength of 23.3 MPa, with a brittle behavior after peak strength. The addition of 0.5% WTTF fibers increased the compressive strength to 25.5 MPa, improved ductility, and enhanced energy absorption capacity (Fig. 11b). These improvements can be attributed to the rough surface texture of the waste tire textile fibers, which leads to a strong bond with shotcrete materials, especially cement. In the shotcrete matrix, the WTTF fibers act as resistance against crack propagation, effectively limiting further damage until the bond between the fiber and cementitious material is lost. However, the compressive strength slightly decreased with 1% WTTF fiber due to increased porosity, and further decreased to 21.4 MPa with 1.5% WTTF fiber (Fig. 11c,d). Despite the decrease in strength, the higher fiber content resulted in significantly higher peak strain and a different failure pattern, with multiple shorter cracks and dilation instead of complete disintegration.

Table 5 summarizes the mean peak strength values obtained. As seen in the table, the strength of shotcrete initially increases and then decreases as the WTTF fiber content increases. Additionally, the table provides information about the strain at peak strength according to the fiber content. There is a direct relationship between the fiber content and the strain at peak strength, meaning shotcrete reinforced with WTTF fibers exhibits greater deformations than fiber-free shotcrete. The deformability of WTTF fibers under compressive loading accounts for this behavior. It is worth mentioning that the WTTF fiber-reinforced shotcrete showed the ability to withstand significant post-break compressive loads and experienced remarkable deformations without complete disintegration.

**Table 5:** Strength and axial strain of peak points vs. WTTFs content.

Sample ID	Fiber content (%)	Peak strength				Axial strain of peak point			
		Compressive strength (MPa)	AVG. (MPa)	SD. (MPa)	COV. (%)	Strain at peak stress (× 10ˉ^3^)	AVG. (MPa)	SD. (MPa)	COV. (%)
S1—0%	0	23.7	23.3	0.54	0.02	6.1	6.97	0.85	0.12
S2—0%		22.7				7.1			
S3—0%		23.5				7.8			
S1—0.5%	0.5	27.3	25.5	1.56	0.06	8.2	7.66	1.03	0.13
S2—0.5%		24.8				8.4			
S3—0.5%		24.5				6.5			
S1—1%	1	21.9	24.7	3.14	0.13	9.1	8.62	0.98	0.11
S2—1%		28.1				7.5			
S3—1%		24.1				9.2			
S1—1.5%	1.5	23.4	21.4	2.37	0.11	6.4	9.95	4.71	0.47
S2—1.5%		22.1				15.3			
S3—1.5%		18.8				8.1			

*AVG* Average value, *SD* Standard deviation, *COV* Coefficient of variation.

**Figure 12:**
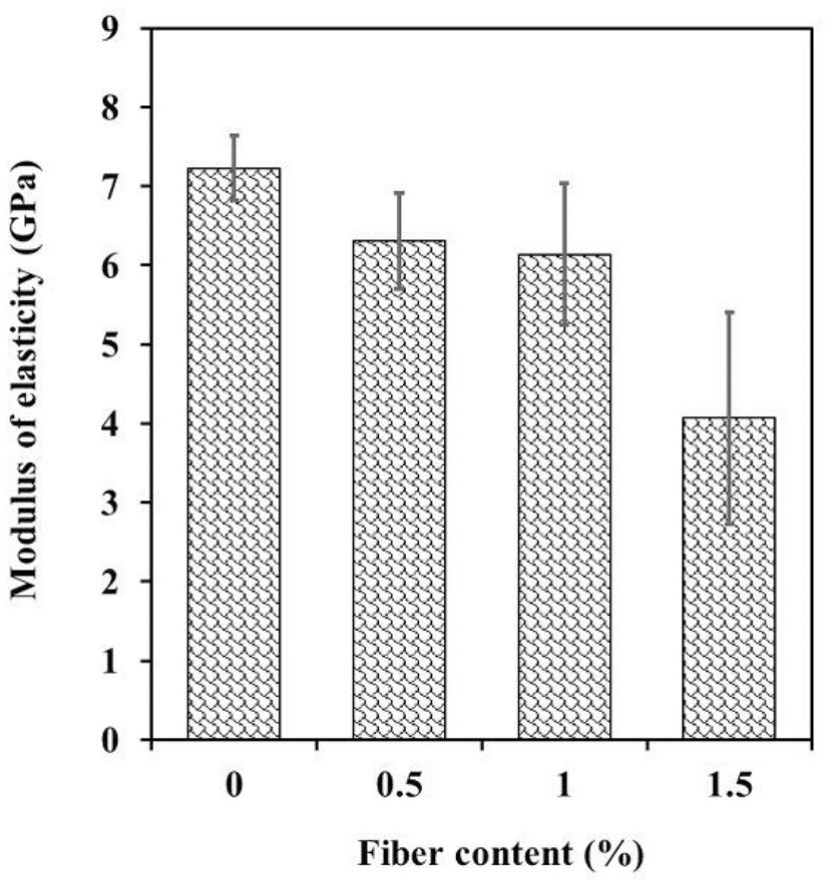
The effect of WTTFs content on modulus of elasticity.

The modulus of elasticity, also known as Young's modulus, was determined by calculating the ratio of the applied stress to the corresponding strain within the elastic limit of the UCS test curves. In Fig. 12, the changes in the modulus of elasticity of the samples are depicted as the WTTF fiber content increases. The graph illustrates that as WTTF fibers are added up to 1.5%, the elastic modulus decreases by more than 43%. This reduction can be attributed to the increased deformability of shotcrete caused by the presence of WTTF fibers. The Young's modulus is influenced by the elastic properties of various components, such as aggregates, cement, and fibers. Therefore, when a higher ratio of WTTF fibers is incorporated into the shotcrete mix, a lower Young's modulus is obtained due to the relatively lower stiffness and Young's modulus of the WTTF fibers compared to the aggregates. Essentially, the addition of WTTF fibers leads to a decrease in the overall stiffness of the shotcrete mixture, resulting in a lower Young's modulus. This indicates that the inclusion of these fibers had an impact on the material's ability to deform elastically under stress, providing insights into its potential structural behavior.

**Figure 13:**
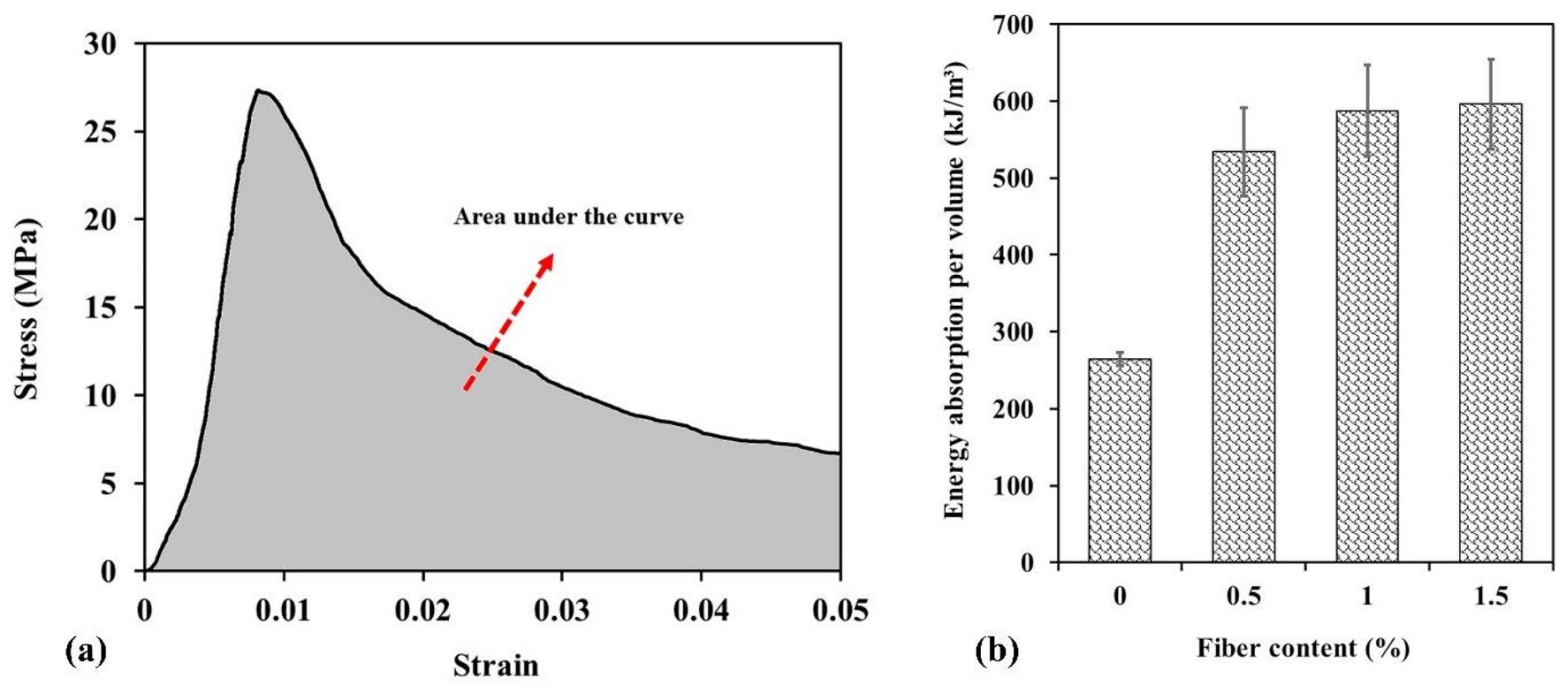
(**a**) Energy absorption capacity calculation, (**b**) energy absorption capacity vs. WTTF fiber content.

The area under the stress–strain curve of the compressive test or load–deflection curve of the flexural test is usually defined as the energy absorption capacity or toughness of the material^12,15,29,73^. In the present study, the area under the stress–strain curve of uniaxial compressive strength tests was considered as energy absorption capacity. For example, in Fig. 13a the area under the stress–strain curve of sample 2 with 0.5% fiber content is presented. Figure 13b shows the average absorbed energy of plain shotcrete specimens and reinforced shotcrete samples with various WTTF fiber contents. As shown in this figure, the energy absorption capacity value of the plain shotcrete samples was determined to be 254 kJ/m^3^, which is very low compared to the energy absorption capacity of WTTF fiber-reinforced shotcrete samples. The energy absorption value of the reinforced shotcrete with 0.5% textile fiber was found to be 534 kJ/m^3^. This means that with an increase of 0.5% of fibers in shotcrete, its energy absorption capacity has increased more than 2 times. As 0.5% of textile fibers were added to the shotcrete samples, the force damping, elastic and plastic deformations, and uniaxial compressive strength increased, and consequently, the energy absorption capacity increased.

**Figure 14:**
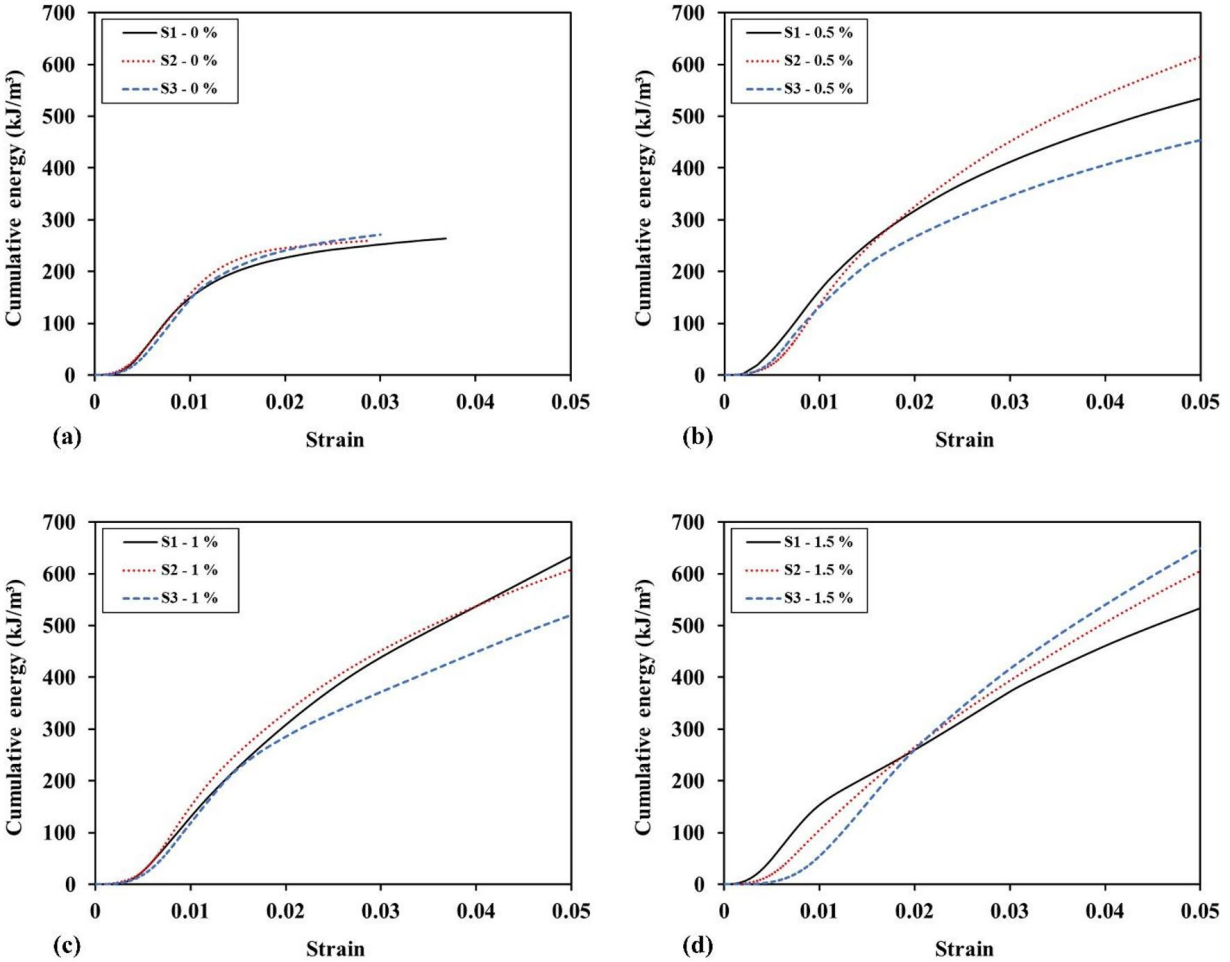
Cumulative energy absorption curves of the samples with different WTTFs ratio.

Figure 14 illustrates the cumulative energy curves per unit volume for all the samples tested. It can be observed that the curves for the fiber-free specimens become almost horizontal after a strain of 0.03, indicating that the samples are broken and unable to absorb more energy. On the other hand, the fiber-reinforced samples are capable of absorbing energy up to higher strains. In this study, the experiments were continued until a strain of 0.05 was obtained.

Furthermore, increasing the fiber content in the shotcrete resulted in an increase in the energy absorption capacity. The samples with 1% and 1.5% WTTF fiber reinforcement exhibited energy absorption capacities of 588 and 596 kJ/m^3^, respectively. Although the energy absorption capacity of the 0.5% fiber-reinforced samples was significantly higher than that of the plain samples, further increases in fiber content (1% and 1.5%) only led to slight improvements in energy absorption capacity. Compared to the 0.5% fiber-reinforced samples, the energy absorbed by the samples increased by 10% and 12% with the addition of 1% and 1.5% WTTF fibers, respectively.

### Splitting tensile strength

**Figure 15:**
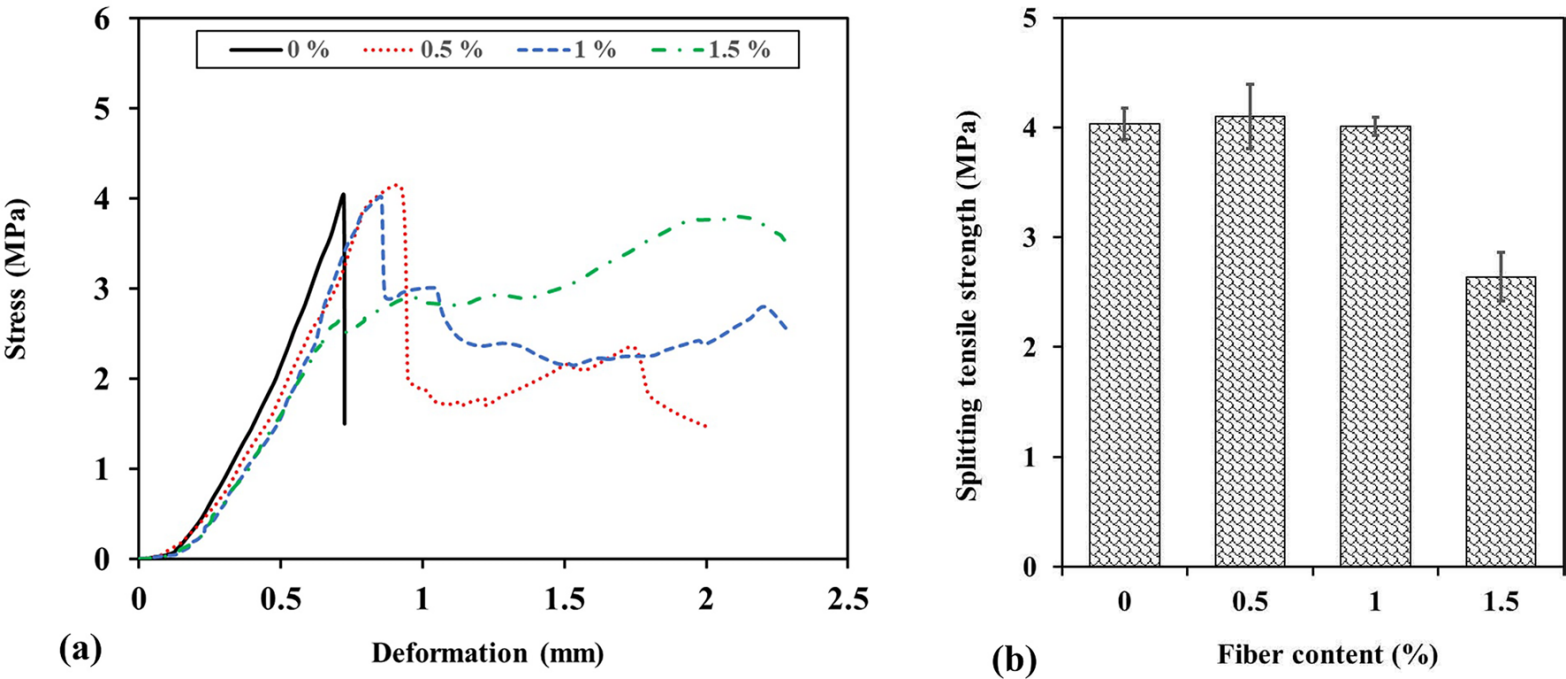
(**a**) Stress-deformation curves of splitting tensile strength tests, (**b**) splitting tensile strength vs. WTTF fiber content.

The stress-deformation curves obtained from splitting tensile strength tests are presented in Fig. 15a. The mechanical behavior of shotcrete samples containing WTTF fibers is significantly different from plain shotcrete, particularly after reaching the peak strength point. Figure 15b shows the splitting tensile strength of shotcrete specimens as a function of the WTTF fiber content. It can be observed that the splitting tensile strength of shotcrete slightly increases with the addition of 0.5% WTTF, but decreases as the fiber content is increased to 1% and 1.5%. This behavior is somewhat similar to the changes seen in uniaxial compressive strength resulting from the addition of fibers.

**Figure 16:**
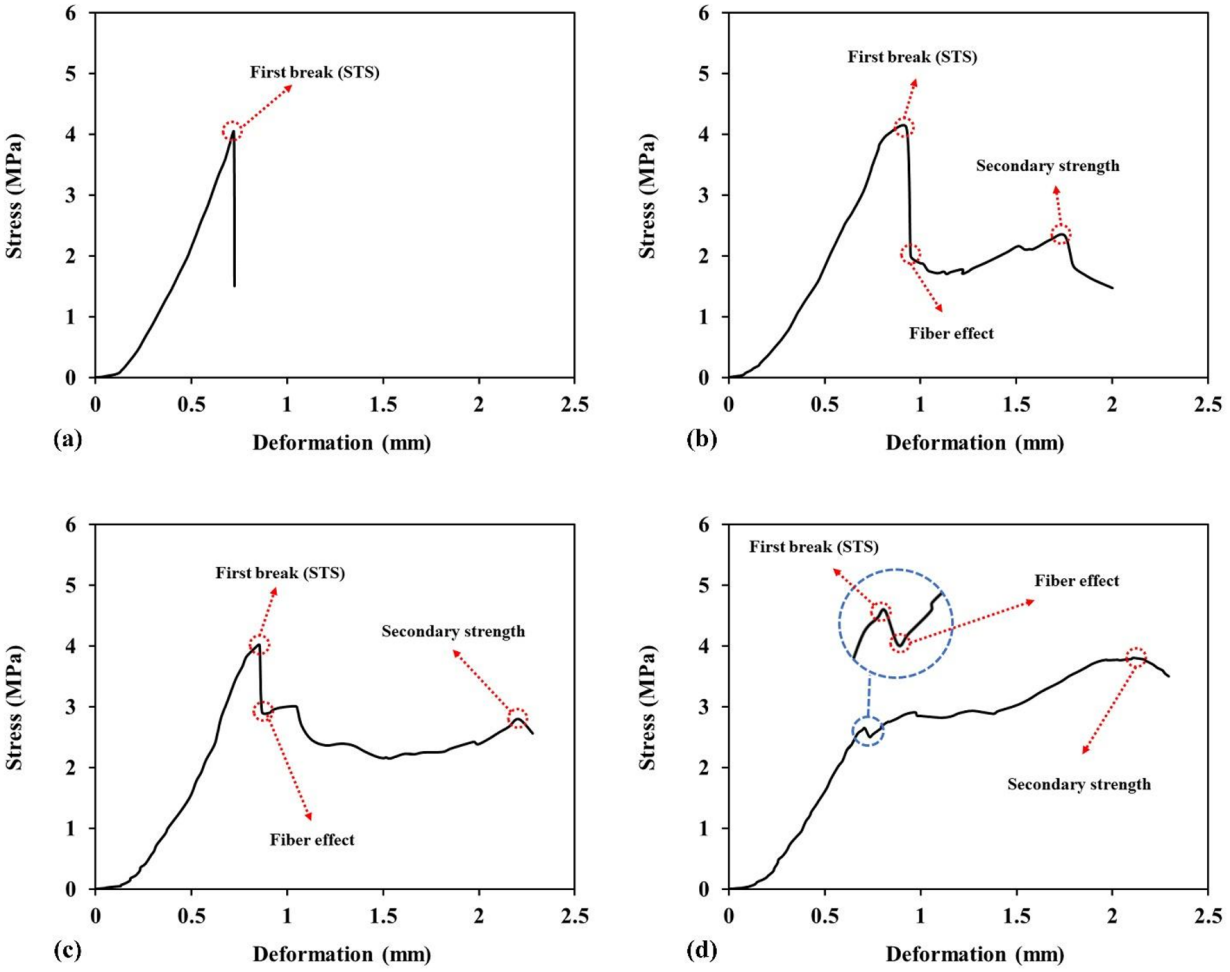
Effect of WTTFs on post-break behaviour of shotcrete samples with: (**a**) fiber-free; (**b**) 0.5% fiber; (**c**) 1% fiber; and (**d**) 1.5% fiber.

The main distinction between the splitting tensile strength curves of WTTF fiber-reinforced shotcrete and fiber-free shotcrete occurs after reaching the peak strength, as illustrated in Fig. 16. Figure 16a shows that the fiber-free shotcrete sample exhibits brittle failure, with the post-break stress abruptly dropping to zero. In contrast, shotcrete samples containing WTTF fiber do not display brittle failure due to the deformable nature of the WTTF fibers. As seen in Fig. 16b, the post-break stress of the sample containing 0.5% fibers decreases to approximately 2 MPa. In other words, the addition of fibers alters the behavior of the shotcrete, allowing the samples to withstand measurable post-break stresses and experience significant deformation even when cracked. This is attributed to the ability of WTTF to undergo considerable deformation before failure, enabling fiber-reinforced samples to exhibit a high capacity for absorbing plastic energy.

**Figure 17:**
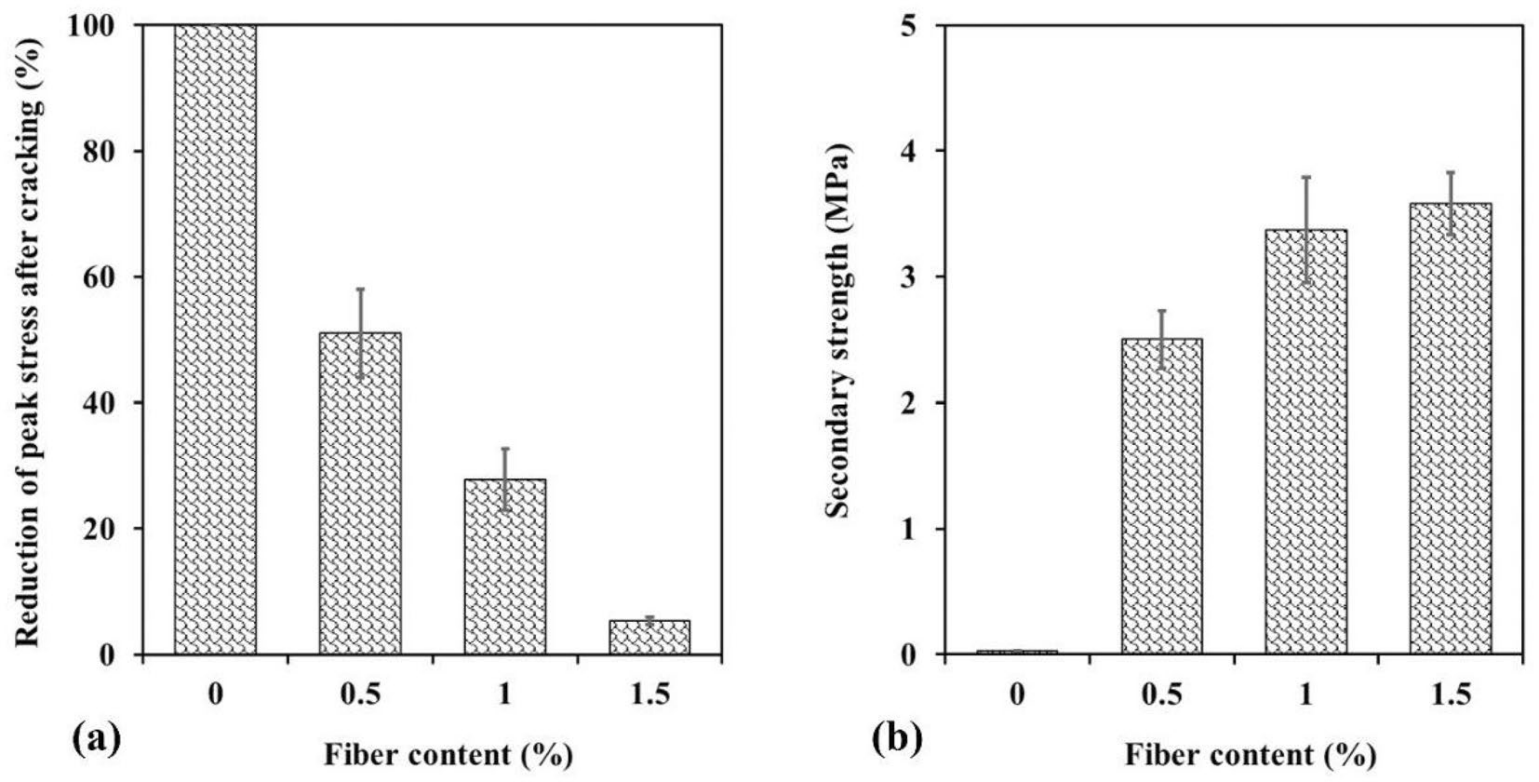
(**a**) Reduction of strength due to first break of samples; (**b**) secondary strength of samples.

Figure 17a illustrates the percentage drop in post-break strength of specimens versus fiber content, indicating that the amount of post-break strength drop decreases with increasing fiber content due to the influence of WTTF. Another observation in the behavior of fiber-reinforced samples is the increase in post-break stresses leading to secondary peak strengths during further deformation, referred to as secondary strength in this study. Figure 17b depicts the secondary strength of the samples as a function of WTTF content. It can be seen that the secondary strength increases with higher fiber ratios. This increase is attributed to the fact that, after initial cracking, the presence of fibers in the sample's matrix prevents rupture from occurring. Consequently, as the fiber content increases, so does the resistance to rupture.

After examining the various impacts of integrating WTTF fibers into the shotcrete mixture, such as their influence on porosity, compressive strength, tensile strength, and energy absorption capacity, it is suggested that a WTTF fiber content ratio of 1% is the most appropriate for utilization in shotcrete. The findings of this research have significant practical implications for the implementation of shotcrete reinforced with waste tire textile fibers in various construction and engineering applications. Firstly, the increase in post-peak behavior and ductility of shotcrete with the addition of up to 1.5% WTTF fibers emphasizes the potential for enhanced performance in absorbing energy and accommodating deformation under loading conditions. The substantial increase in energy absorption capacity, as well as the sustained post-break stresses and considerable deformation after cracking, demonstrate the potential for improved resilience and structural integrity when utilizing WTTF fibers in shotcrete mixtures. Furthermore, the reduction of dry density by adding WTTF fibers improves the quality of shotcrete operation in the roof and walls of underground spaces such as tunnels. Moreover, the study's recommendation of a 1% WTTF fiber content ratio as the most appropriate for utilization in shotcrete serves as a practical guideline for engineers and practitioners seeking to leverage the benefits of fiber reinforcement while balancing factors such as porosity, compressive strength, and energy absorption capacity. Additionally, considering the adverse environmental and health effects associated with WTTF fibers, their recycling and utilization in shotcrete applications offer not only economic advantages but also crucial environmental benefits, contributing to the conservation of natural resources. This underscores the multifaceted value of exploring WTTF fibers as a sustainable alternative in the field of shotcrete technology.

## Conclusion

This study focused on examining the physical and mechanical characteristics of shotcrete that had been reinforced with different proportions of waste tire textile fibers. The research aimed to assess how the inclusion of these fibers impacted the overall performance and properties of the shotcrete material. Upon conducting a series of tests and analyses, several significant findings were obtained from the study:

- Addition of WTTF fibers to shotcrete at a content of up to 1.5 percent resulted in a reduction of approximately 10% in dry density. The reason for this reduction is the lower WTTF fiber density compared to other components of shotcrete, including aggregate.
- The addition of WTTF fiber content notably influenced water absorption. Incorporating 1.5% WTTF fibers resulted in a substantial increase of approximately 43% in water absorption. This rise in water absorption can be attributed to the formation of increased void volume between WTTF fibers and the cement paste within the shotcrete mixture.
- The porosity of fiber-free shotcrete was measured at 14.87%. However, as the WTTF fibers were gradually increased up to 1.5%, the porosity experienced a notable increase of approximately 30%, resulting in a final measurement of 19.35%. This increase in porosity can be attributed to the incorporation of WTTF fibers, which contribute to the formation of additional voids within the shotcrete material.
- Shotcrete mixtures with higher WTTF fiber incorporation exhibited lower ultrasonic pulse velocities. Specifically, the ultrasonic pulse velocity decreased by 1.9%, 9.5%, and 13.4% as the fiber content increased by 0.5%, 1%, and 1.5%, respectively. This decrease was primarily attributed to the development of voids caused by the addition of WTTF fibers.
- The presence of WTTF fibers minimally affected the uniaxial compressive strength of shotcrete, but significantly altered its post-peak behavior, making it more ductile and capable of absorbing more energy during loading. So that with the increase of 0.5% of fibers in shotcrete, its energy absorption capacity has increased from 254 to 534 kJ/m^3^, which means more than 2 times increase.
- The addition of WTTF fibers has led to a decrease in the overall stiffness of the shotcrete mixture, resulting in a lower Young's modulus. So that with the addition of WTTF fibers up to 1.5%, the elastic modulus decreased by more than 43%.
- In the splitting tensile test, plain shotcrete failed abruptly at the ultimate strength, whereas WTTF-reinforced shotcrete showed sustained post-break stresses and considerable deformation after cracking due to the ability of WTTF fibers to undergo substantial deformation before failure, contributing to high energy absorption capacity.
- After examining the various impacts of integrating WTTF fibers into the shotcrete mixture, it is suggested that a WTTF fiber content ratio of 1% is the most appropriate for utilization in shotcrete.

In summary, the findings of this study highlight the substantial influence of WTTF fibers on the mechanical performance of shotcrete, underscoring their potential as viable replacements for synthetic fibers traditionally used in the shotcrete industry.

## Data Availability

The datasets generated and analyzed during the current study available from the corresponding author on reasonable request.
